# Years worked at night and body mass index among registered nurses from eighteen public hospitals in Rio de Janeiro, Brazil

**DOI:** 10.1186/s12913-014-0603-4

**Published:** 2014-11-29

**Authors:** Rosane Härter Griep, Leonardo S Bastos, Maria de Jesus Mendes da Fonseca, Aline Silva-Costa, Luciana Fernandes Portela, Susanna Toivanen, Lucia Rotenberg

**Affiliations:** Laboratory of Health and Environment Education, Oswaldo Cruz Institute, Oswaldo Cruz Foundation (Fiocruz), Av. Brasil 4365, Manguinhos, 21040-360 Rio de Janeiro, RJ Brazil; Scientific Computing Program (PROCC), Fiocruz, Rio de Janeiro, RJ Brazil; National School of Public Health, Fiocruz, Rio de Janeiro, RJ Brazil; Centre for Health Equity Studies (CHESS), Stockholm University, Karolinska Institutet, Stockholm, Sweden

**Keywords:** Nurse, Public hospitals, Body mass index, Night work, Shift work

## Abstract

**Background:**

Employees working night shifts are at a greater risk of being overweight or obese. Few studies on obesity and weight gain analyze the years of exposure to night work. The aim of this study was to determine the relationship between the years of exposure to night work and body mass index (BMI) among registered nurses.

**Methods:**

A cross-sectional analysis was performed in 18 largest public hospitals in Rio de Janeiro, Brazil. A total of 2,372 registered nurses (2,100 women) completed a comprehensive questionnaire concerning sociodemographic, professional, lifestyle, and health behavioral data. Current and past exposures to night shifts as well as BMI values were measured as continuous variables. A gamma regression model was used with an identity link function to establish the association.

**Results:**

The association between years of exposure to night work and BMI was statistically significant for both women and men after adjusting for all covariates [β = 0.036; CI95% = 0.009–0.063) and β = 0.071 (CI95% = 0.012–0.129), respectively]. The effect of night work was greater among men than women. For example, for those women who have worked at night for 20 years the estimated average BMI was 25.6 kg/m^2^ [range, 25.0–26.2]. In relation to men, after 20 years of exposure to night work the estimated average BMI was 26.9 kg/m2 [range, 25.6–28.1].

**Conclusions:**

These findings suggest that night shift exposure is related to BMI increases. Obesity prevention strategies should incorporate improvements in work environments, such as the provision of proper meals to night workers, in addition to educational programs on the health effects of night work.

## Background

Overweight and obesity have complex and multifactorial etiologies and have reached global epidemic proportions [1]. As an indirect result of the population getting fatter, health care costs are thought to be increasing, including indirect costs due to disability pensions and loss of productivity [2]. Overweight and obesity also lead to an elevated risk of some forms of cancer [3], cardiovascular, digestive diseases, diabetes mellitus, sleep apnea, osteoarthritis [4], and all-cause mortality [4,5].

Shift work and atypical working hours, which have increased substantially in recent decades [6], have been increasingly addressed in the international literature due to their associated cardiovascular risks, which include obesity and other metabolic syndrome symptoms [7]. Changes in nutrition, smoking, alcohol consumption, and exercise due to unusual working hours can increase the risk of obesity among shift workers [8]. In addition, shift work is associated with poor sleep [9], which increases appetite and unhealthy snacking [10,11]. Reduced sleep and physical activities, changed eating habits and patterns, and circadian rhythm disturbances are postulated as mediator mechanisms to explain the metabolic disturbances resulting from shift work [12,13].

Some studies on shift work and weight have shown a higher prevalence of obesity in night shift workers than in day shift workers [14-18], although other studies do not show such associations [19-21]. In a recent review, van Drongelen et al. [7] found strong evidence for crude association, which become non-significant when confounders have been controlled for. So, the authors concluded that there is insufficient evidence for the link between shift work exposure and weight gain.

Another inconsistency in the literature concerning the link between shift work and body mass index (BMI) is related to gender differences. Two recent studies reported that night shift schedules were associated with an increase in BMI among female but not male nurses [22,23]. Nevertheless, a longitudinal study dealing exclusively with men showed that rotating shift work was an independent risk factor for weight gain [18].

Few studies on obesity and weight gain analyze the years of exposure to night work [24-26]. This approach seems particularly suitable for studies on this topic, since obesity develops over long periods. Another improvement in this area of studies is the analysis of night work history, such as whether the person worked at night previously and for how long. This information has been used in investigations on the influence of night work on health, including aspects related to obesity [26]. Night work history permits the study of former night workers, which has proven to be a fruitful approach in studies on lifestyle, habits, and health, including obesity and sleep disturbances [26].

The present paper deals with BMI of female and male nurses and considers their occupational history as regards work schedules. The aim of this study was to determine the relationship between the years of exposure to night work and BMI and to examine related gender differences among registered nurses.

## Methods

### Study population

This cross-sectional study was performed in the 18 largest public hospitals in the city of Rio de Janeiro, Brazil. The eligible group was comprised of registered nurses providing assistance to patients at the hospitals. The nurses were invited to participate through a face-to-face approach by a team of interviewers. Of a total of 3,904 eligible nurses, 3,229 (82.7%) returned completed questionnaires.

### Data collection

Data collection took place from March 2010 to November 2011. Data were collected during work hours at the hospitals studied, through a comprehensive self-reported questionnaire that provided detailed information about the nursing job and health and socioeconomic conditions. The questionnaire was submitted to five rounds of pretesting (*n* = 50) to improve the clarity of the survey items.

A team of trained interviewers explained the objectives of the study, obtained written consent, and explained how the questionnaire was to be completed. Participants completed the questionnaire, which included questions on weight (both current weight and that at age 20). Additionally, the interviewers scheduled a date with participants for the return of the completed questionnaires.

### Measures

In most Brazilian hospitals, continuous work along 24 hours is provided by a shift system with 12 hours at daytime (7:00 am to 7:00 pm) or at night (7:00 pm to 7:00 am), followed by either 36 or 60 hours off. There are also schemes which involve only work on week days, either in the morning, or in the afternoon or both morning and afternoon. These working hours make it possible to engage in more than one professional employment most commonly something in the field of health care. Therefore, the definition of workers’ schedule has to consider all jobs he/she is engaged in.

### Exposure variable: years worked at night

This variable was defined as the number of complete years of work at night shifts. A night worker was defined as one who had at least one night job on the occasion of the research, *i.e.,* one who worked at night at least once a week or 4 times a month in 12-h shifts. Day workers were asked “Have you ever worked nights?” Possible answers were: (1) Yes, regularly, once a week, (2) Yes, regularly, two to three times a week, (3) Yes, regularly, four or more times a week, (4) Yes, rarely, (5) Yes, occasionally, (6) No. Those who answered “1”, “2” or “3” were classified as “former night workers”, whereas those answering “4”, “5” or “6” were classified as “day workers with no experience in night work. Current and former night workers were asked, respectively: “How long have you been working at night in nursing, here or in another place?” and “For how long did you work at night?” Those who had never worked at night and those who had worked at night for less than or equal to 1 year were considered to have “zero” years of nightshift.

### Outcome measure: BMI

BMI was calculated as weight (kg)/height (m)^2^ and was used as a continuous variable in analysis.

### Covariates

The socio-demographic covariates were gender, age in years (continuous), marital status (married and not married), and *per capita* income in USD, considering the conversion rate at the time of data collection (continuous). Besides, weekly work hours (continuous), alcohol consumption, smoking (never smoker, ex-smoker and current smoker), leisure-time physical activity (yes and no), usual sleep duration (continuous) and self-reported BMI at 20 years old (continuous), were used as covariates.

Weekly work hours were defined as the time devoted to professional work based on the question “Now let’s recall the hours that you dedicated to professional nursing each day of last week (at all places)”*.* Participants recorded the actual time they arrived at and left the hospital on a daily basis, regardless of official work schedules. Test-retest reliability, as measured by the intraclass correlation coefficient, was 0.68 (95% confidence interval, 0.50–0.80), as reported in a previous study on nursing workers [27].

Alcohol consumptions were quantified based on common measures (glass, can, bottle, and dose). The doses were as follows: beer (200 ml or a double glass), wine (150 ml or a cup); and spirits/distilled (50 ml or one measured amount). Alcohol consumption was classified into four categories as follows: “abstains from alcohol consumption”, “low consumption” (<4 doses/month), “medium consumption” (5 to 7 doses up to 4 times/month), and “high consumption” (≥8 doses more than 2 times/week).

Self-reported BMI at 20 years old was based on height at study entry and self-reported recall of weight at age 20.

### Statistical analysis

Analyses were conducted separately for women and men. A chi-square test was used to study descriptive analyses of sociodemographic variables as well as other variables related to work, whereas analysis of variance (ANOVA) was used for continuous variables. Differences were considered significant at *p* <0.05. To facilitate the visualization and interpretation of univariate associations, the variable *years* of *work at night* was categorized into three levels (<1 yr, 1–9 years; 10+ years). However, in the statistics modeling both past and present exposures to night shifts as well as BMI were measured as continuous variables so as to avoid loss of information [28]. A gamma regression model with an identity link function was used for multivariate analysis [29]. Residual analysis was performed to test the adequacy of the model. Age, BMI at the age of 20, *per capita* income, marital status, and number of biological children were considered adjustment variables. Usual sleep duration, leisure-time physical activity, smoking, and alcohol consumption were considered mediator mechanisms to explain the relationship between night shift work and BMI, as described by literature [30]. Despite results from interaction models, that did not indicate a significant influence of sex on the association between years of work at night shift and BMI (p = 0.752), analyses were stratified by sex considering (1) that different factors favor weight gain for men and women [31-34], and (2) the evidences of gender-differences in the relationship between shift schedule and BMI [22,23]. This procedure considered that, on the one hand, the low number of men in this study compared to women leads to an unavoidable limitation in statistical power in all results on men, but, on the other hand, controversies on gender differences justify gender-stratified analysis.

The final model for each gender was chosen by Akaike Information Criterion (AIC). Predictions for the BMI at different years of exposure to work were made from the final model, considering all covariates (mean centered continuous variables: age, BMI at age of 20 years, per capita income, weekly work hours, and usual sleep duration, and categorical variables fixed in low risk: not married, without biological children, no smoking, no alcohol consumption, and physically active in leisure time). Data were analyzed by using the free software R, version 2.15 (R Development Core Team, Vienna, Austria).

### Ethical considerations

The study was briefly explained to participants, who were informed that involvement was completely voluntary and that they could withdraw at any time with no negative consequences. All participants signed consent forms. The protocol was submitted and accepted by the Oswaldo Cruz Foundation (Fiocruz) Ethics Research Committee.

## Results

From the 3,229 current nurses in the study, 857 (26%) had missing information in one or more analysis variable. The remaining 2,372 individuals (2,100 women) were included in the analyses. No differences were observed between the analyzed group and individuals with missing information in sociodemographic, occupational, or behavioral variables. The average number of years of worked at night for men and women were 10.82 (SD = 9.27) years and 6.84 (SD = 6.95) years, respectively. Moreover, the proportion of workers with different years worked at night varied between sexes. Among men, 12.1% had never worked at night, 38.9% had worked between 1 and 9 years, and 48.9% had worked 10 or more years. Among women, these proportions were 21.3%, 48.0%, and 30.7%, respectively.

The bivariate analysis of selected variables and years worked at night are shown in Tables 1 and 2. Women who had done 10 or more years of night work were more likely to be older, have a higher *per capita* income, work more hours per week, have a higher current BMI, and be current smokers than those without night work experience (Table 1). Men who had worked at night for many years were older, had a higher current BMI, and were less frequently married than men with no experience in night shift work (Table 2).

**Table 1:** **Demographic, occupational, health, and work-related psychosocial characteristics of study participants and bivariate associations between these factors and years worked at night among women (** ***n*** **= 2,100)**

	**All (** ***n*** ** = 2,100)**	**Years worked at night**			***p*** **-value**
		**<1 (** ***n*** ** = 447)**	**1 to 9 (** ***n*** ** = 1,008)**	≥**10 (** ***n*** ** = 645)**	
Age, mean (SD)	39.5 (9.8)	38.2 (10.9)	36.6 (9.1)	44.9 (7.5)	<0.001
*Per capita* income (USD)^a^, mean (SD)	1,061.1 (709.2)	1,001.6 (680.5)	1,053.9 (689.2)	1,113.6 (755.3)	0.033
Weekly hours worked, mean (SD)	54.2 (19.1)	47.8 (17.5	54.4 (18.8)	57.8 (19.3)	<0.001
Years at work in nursing, mean (SD)	15.2 (9.7)	15.18 (9.8)	15.2 (9.6)	15.6 (9.6)	0.697
BMI at 20 years, mean (SD)	21.1 (3.3)	21.1 (3.2)	21.2 (3.4)	21.1 (3.4)	0.604
BMI (current), mean (SD)	26.1 (5.1)	25.4 (4.8)	25.7 (4.9)	27.2 (5.4)	<0.001
Number of biological children, mean (SD)	0.97 (1.0)	0.80 (0.94)	0.88 (0.99)	1.23 (1.03)	<0.001
Marital status (% married)	58.9	56.8	59.8	58.9	0.563
Smoking status					
Never smoker	76.6	77.7	80.8	69.2	<0.001
Ex-smoker	15.0	15.0	11.7	20.3	
Current smoker	8.4	7.4	7.5	10.5	
Alcohol consumption					
Abstained	38.9	41.6	40.9	34.0	0.083
Low	20.0	19.2	18.8	22.6	
Medium	32.2	32.1	32.8	34.4	
High	7.9	6.9	7.5	9.0	
Physical activity (% yes)	30.2	32.4	30.0	29.0	0.464
Sleep time, mean (SD)	6.9 (1.5)	6.9 (1.5)	6.9 (1.5)	7.0 (1.6)	0.235

^a^Price on 12/30/2012 (BRL2.02).

**Table 2:** **Demographic, occupational, health, and work-related psychosocial characteristics of study participants and bivariate associations between these factors and years worked at night shift among men (** ***n*** **= 272)**

	**All (** ***n*** ** = 272)**	**Years worked at night**			***p*** **-value**
		**<1 (** ***n*** ** = 33)**	**1 to 9 (** ***n*** ** = 106)**	≥**10 (** ***n*** ** = 133)**	
Age, mean (SD)	41.3 (10.2)	36.2 (10.1)	35.1 (7.9)	47.5 (8.0)	<0.001
*Per capita* income (USD)^a^, mean (SD)	1,073.7 (743.6)	909.7 (611.2)	1,105.1 (762.2)	1,132.3 (755.4)	0.283
Weekly work hours, mean (SD)	59.3 (18.7)	54.2 (20.2)	58.7 (18.7)	61.0 (18.3)	0.166
Years of work in nursing, mean (SD)	14.3 (9.5)	14.2 (9.7)	13.7 (9.1)	14.7 (9.7)	0.744
BMI at 20 years, mean (SD)	22.4 (3.0)	22.7 (2.7)	22.8 (3.1)	22.1 (3.1)	0.217
BMI (current), mean (SD)	27.3 (4.1)	26.4 (3.4)	27.0 (3.9)	27.9 (4.3)	0.099
Marital status (% married)	69.1	54.5	62.3	78.2	0.005
Smoking status					
Never smoker	72.8	78.8	74.5	69.9	0.596
Ex-smoker	18.8	18.2	18.9	18.8	
Current smoker	8.4	3.0	6.6	11.3	
Alcohol consumption					
Abstained	26.8	30.3	27.4	25.6	0.145
Low	18.0	12.1	14.2	22.6	
Medium	40.1	54.5	39.5	36.8	
High	15.1	3.1	18.9	15.0	
Physical activity (% yes)	41.2	45.5	43.4	38.3	0.636
Usual sleep duration, mean (SD)	6.9 (1.6)	6.8 (1.5)	7.0 (1.7)	6.9 (1.6)	0.709

^a^Price on 12/30/2012 (BRL2.02).

Adjusted regression models (models 2–6 in Tables 3 and 4) found positive, independent associations between years worked at night and higher BMI levels for both women and men. Among women, the association was statistically significant (β =0.107 [CI95% = 0.074–0.139], model 1 in Table 3) and was still significant after adjustment for all covariates (β =0.036 [CI95% = 0.004–0.063], model 6 in Table 3). We found some evidence that associations between the years worked at night and BMI are mediated at least in part through health behavior (Table 3). The mean change in BMI decreased from β =0.040 [CI95% = 0.013–0.067] in model 5 (controls for age, BMI at 20 years old, marital status, *per capita* income, number of biological children, and weekly work hours) to β =0.036 [CI95% = 0.008–0.063] in model 6 (which additionally controls for smoking, alcohol consumption, and physical activity) (Table 3). Similar results were observed in men: β =0.066 (CI95% = 0.015–0.118, model 1 in Table 4) and β =0.079 (CI95% = 0.019–0.138, model 6 in Table 4), before and after model adjustment, respectively. However, the influence of health behavior was not detected in men.

**Table 3:** **Multiple linear regression analysis of years worked at night work on BMI among women (n=2,100)**

**Models**	**Beta**	**95% CI**	**p-value**	**AIC**
Model 1 – Unadjusted	0.107	0.074–0.139	<0.0001	12249.2
Model 2 – Model 1 + age + years at work	0.061	0.026–0.095	0.0006	12163.8
Model 3 – Model 2 + BMI at 20 years old	0.045	0.018–0.072	0.0010	11236.0
Model 4 – Model 3 + marital status + per capita income + number of children	0.046	0.019–0.072	0.0008	11199.0
Model 5 – Model 4 + weekly hours worked	0.040	0.013–0.067	0.0040	11197.5
Model 6 – Model 5 + smoking, alcohol consumption, physical activity, and usual sleep duration	0.036	0.009–0.063	0.0087	11161.0

**Table 4:** **Multiple linear regression analysis of the years worked at work on BMI among men (** ***n*** **=272)**

**Models**	**Beta**	**95% CI**	***p*** **-value**	**AIC**
Model 1 – Unadjusted	0.066	0.015–0.118	0.0128	1479.1
Model 2 – Model 1 + age + years at work	0.066	−0.002–0.134	0.0593	1479.3
Model 3 – Model 2 + BMI at 20 years old	0.076	0.018–0.134	0.0116	1399.8
Model 4 – Model 3 + marital status + *per capita* income	0.072	0.013–0.131	0.0180	1402.2
Model 5 – Model 4 + weekly hours worked	0.065	0.006–0.124	0.0320	1400.8
Model 6 – Model 5 + smoking, alcohol consumption, physical activity, and usual sleep duration	0.071	0.012–0.129	0.0187	1398.1

Model 6 in Tables 3 and 4 shows the final model for men and women according to AIC. We observed that the effect of night work on BMI was greater in men, 0.071 kg/m^2^ per year of night work [range 0.012–0.129]. The estimated effect of night work for women was 0.036 kg/m^2^ per year of night work [range 0.009–0.063] (Tables 3 and 4).

**Figure 1:**
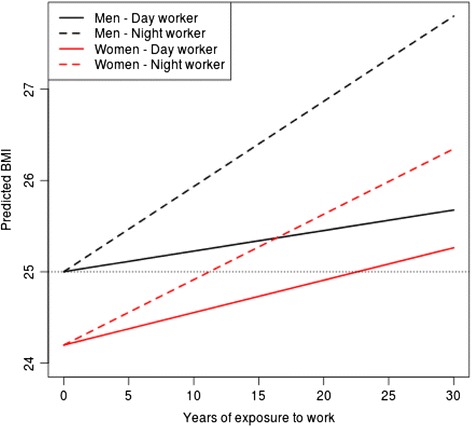
**Predicted BMI and years of exposure to work by gender.** Note that any individual with a BMI above 25 (the dotted line) is considered to be overweight.

For the final model, Figure 1 presents the predicted BMI over years of exposure to work by gender. The data show that the years of exposure to night work increases the BMI. The effect of night work was greater among women than men. The estimated coefficient of exposure to night work for the women was 0.035 [range 0.019–0.052] and 0.022 [range −0.020–0.066] for the men. However, men were more affected by exposure to night work than women. For example, the estimated average BMI was 24.2 kg/m^2^ [range, 23.7–24.7] for those women who did not work at night and 24.3 kg/m^2^ [range, 23.8-24.8] for those who worked at night for 1 year. For those women who worked at night for 20 years, the estimated average BMI was 25.6 kg/m^2^ [range, 25.0–26.2]. In relation to men, considering the same adjustment, except with the inclusion of the biological children variable, the estimated average BMI was 25.0 kg/m^2^ [range, 23.7–26.4] for those who did not work at night and 25.1 kg/m^2^ [range, 23.8–26.4] for those who worked at night for 1 year. After 20 years of exposure to night work the estimated average BMI was 26.9 kg/m^2^ [range, 25.6–28.1].

## Discussion

The current study used a self-reported questionnaire to investigate the association between night work and BMI in Brazilian nurses. The significant association found between the years worked at night and the BMI increase for both women and men after adjustment for confounders suggests that the cumulative exposure to night work plays a role in weight gain. Our results corroborate the concept that obesity develops over long periods [35] and, consequently, confirms the relevance of cumulative exposure to night work over time in analysis of this issue.

The mechanisms linking shift/night work to obesity are still unclear, but the proposed pathways include reduced leisure-time physical activity, increased alcohol consumption, difficulty in maintaining a healthy diet or increased consumption of energy-dense foods to combat fatigue, and reduced amount and/or quality of sleep [31,36-38]. Poor sleep is associated with poorer diet and reduced exercise [38]. In addition, clinical investigations of night workers highlight the relationship between circadian rhythms and metabolic homeostasis. Therefore, circadian misalignment due to night work influences hormone release, nutrients, and meal times, all of which have implications for obesity [31]. Lowden et al. [36] highlight the difficulties in absorbing nutrition during the night because of day oriented circadian.

The negative physiological effects of night work may result not only from the type of nutrition, but also from the timing of eating in relation to the 24-h cycle. Night workers tend to have more irregular nutrition patterns than day workers and usually have a larger number of meals, consuming snacks during the night shift [39]. The nutritional habits and quality of meals of nurses who work at night may be related to the ingestion of easily prepared food and snacks used as a strategy to combat sleepiness. Furthermore, ponderal gain may be a consequence of a lack or reduction of physical activity, especially on the day after the night shift [32], due to sleep and physical and mental fatigue.

Specific determinants can explain the relationships between features of work environment and weight gain for men and women. In the present study, the associations between the years of exposure to night work and BMI were attenuated after adjustment for smoking, alcohol consumption, and physical activity among women, but not among men. Although the presence and strength of the mediating relationship cannot be adequately examined in observational and cross-sectional analyses, these results are compatible with the postulated mediating role of these behaviors [29]. These findings may partly be related to the higher exposure to night work among men than women (e.g., the proportions of male and female nurses who worked night shifts for more than 10 years were 48.9% and 30.7%, respectively). It is also possible that night work affects weight gain more intensively in men. However, the small size of the male sample may have influenced our findings, leading to a lack of statistical power to detect the influence of covariates on the association between the years worked at night and BMI.

It is important to highlight that the behavior pattern of Brazilian male nurses differs from that of female nurses, not only regarding night shifts [34], but also in the relationship between aspects of work and behavior [40]. Previous data from the same sample showed that the long weekly work hours are associated with a greater consumption of fried food and coffee, to less physical activity, and to being overweight and obese among female nurses, whereas among male nurses only physical activity is associated with weekly work hours [40].

A review on gender differences in obesity [41] emphasizes that the causes of obesity, both biological and social, vary according to gender. The authors highlight that although it is recognized that biological differences are related to specific sex patterns in weight gain, disparities related to gender and sociocultural factors are still absent from public policy speeches addressing obesity, rendering public policy approaches insufficient when it comes to potential solutions. Studies that explore the influence of night work on behavior and biological aspects could elucidate the mechanisms that relate the different patterns of weight gain between men and women that work at night.

The approach used here is in accordance with the concept that weight gain happens gradually and that night work experience should be considered, even in those who currently work during the day. Although we followed a transversal design, we were able to explore the cumulative exposure to night work during professional life from the night work history. This procedure may explain the differences observed in relation to other studies on obesity such as, for example, the value of beta, which was much inferior to that described by Marqueze et al. [25] and van Amelsvoort et al. [24]. In the present study, as the data were analyzed as a whole for the entire sample (both sexes), workers without experience in night work (whose exposure time was therefore zero) may have contributed to the reduction in the tendency of the curves. The significance of the associations for men and women, even with the inclusion of workers without exposure to night work, suggests the relevancy of years of exposure to night work on obesity.

Since we consider that cumulative exposure to night shift over time is likely to be more relevant than the exposure at a given time, comparison between those who work day and night shifts was avoided. This procedure differs from the usual one in this area, with many studies focusing on current work schedule, comparing day workers to night workers [22] or day workers to rotating shift workers [17]. It is important to highlight that, in the absence of information on occupational history, one cannot exclude the possibility that the group of day workers include people with previous night or shift experience, which may reduce the differences between groups being compared, given the problems that manifest in former night workers [26].

The particularities of excessive workloads should be considered in the generalization of our findings to other contexts. In Brazilian hospitals, nurses usually work 12-h shifts followed by 36 h or 60 h off [32,42]. Working nonconsecutive days, in combination with a low salary, favor the engagement in more than one nursing job [27]. The long working weeks are particularly relevant among night workers [32] and can lead to exhaustion and fatigue due to insufficient recovery time [42,43], possibly affecting workers’ health. For example, whereas the weekly work load of nurses was over 50 h, the average work load for nurses of European countries varied from 24.8 h/week in the Netherlands to 38.5 h/week in Poland [44].

Some methodological limitations may have influenced our results. Although we were able to obtain data on night work history, the same was not possible in relation to the behavioral variables. Besides, eating habits, considered a relevant mediating factor between shift work and obesity [30], were not included in the present analyses.

It is worth mentioning that two variables related to the past—weight at 20 years old and night work for both current and past night workers—could have been affected by worker’s recollections. Nevertheless, we should not suppose that the ability to recall past exposure was dependent on exposure/outcome categories, which would characterize a recall bias. The test-reliability of the reported weight at age 20 was excellent (Intraclass correlation coefficients above 0.90) for men and women in another Brazilian study [45]. Besides, we included both self-reported weight and self-reported height to calculate BMI. Although the variables might have been affected by differential reporting error, the good agreement and validity for self-reported BMI has already been shown [45-47].

## Conclusions

The results of this study suggest that night work plays a role in increases in BMI. Further studies are needed to better understand the biological mechanisms involved and the complex behavioral and social challenges experienced by night shift workers. As night work is essential to some professions, prevention strategies of obesity should incorporate improvements in the work environment, such as the provision of proper meals to night workers, as well as educational programs on the health effects of night work. Finally, we must consider the need for careful examination of legislation to reduce exposure to night work, with a guarantee of changing work schedules, to minimize the unavoidable consequences of night work to health.
